# Prioritizing genes associated with prostate cancer development

**DOI:** 10.1186/1471-2407-10-599

**Published:** 2010-11-02

**Authors:** Ivan P Gorlov, Kanishka Sircar, Hongya Zhao, Sankar N Maity, Nora M Navone, Olga Y Gorlova, Patricia Troncoso, Curtis A Pettaway, Jin Young Byun, Christopher J Logothetis

**Affiliations:** 1 Department of Genitourinary Medical Oncology, The University of Texas MD Anderson Cancer Center, Houston, Texas; 2 Department of Pathology, The University of Texas MD Anderson Cancer Center, Houston, Texas; 3 Department of Epidemiology, The University of Texas MD Anderson Cancer Center, Houston, Texas; 4 Department of Urology, The University of Texas MD Anderson Cancer Center, Houston, Texas; 5 Institute of Advanced Computing and Digital Engineering, Shenzhen Institutes of Advanced Technology, Chinese Academy of Sciences, Shenzhen, China

## Abstract

**Background:**

The genetic control of prostate cancer development is poorly understood. Large numbers of gene-expression datasets on different aspects of prostate tumorigenesis are available. We used these data to identify and prioritize candidate genes associated with the development of prostate cancer and bone metastases. Our working hypothesis was that combining meta-analyses on different but overlapping steps of prostate tumorigenesis will improve identification of genes associated with prostate cancer development.

**Methods:**

A *Z *score-based meta-analysis of gene-expression data was used to identify candidate genes associated with prostate cancer development. To put together different datasets, we conducted a meta-analysis on 3 levels that follow the natural history of prostate cancer development. For experimental verification of candidates, we used in silico validation as well as in-house gene-expression data.

**Results:**

Genes with experimental evidence of an association with prostate cancer development were overrepresented among our top candidates. The meta-analysis also identified a considerable number of novel candidate genes with no published evidence of a role in prostate cancer development. Functional annotation identified cytoskeleton, cell adhesion, extracellular matrix, and cell motility as the top functions associated with prostate cancer development. We identified 10 genes--*CDC2, CCNA2, IGF1, EGR1, SRF, CTGF, CCL2, CAV1, SMAD4*, and *AURKA*--that form hubs of the interaction network and therefore are likely to be primary drivers of prostate cancer development.

**Conclusions:**

By using this large 3-level meta-analysis of the gene-expression data to identify candidate genes associated with prostate cancer development, we have generated a list of candidate genes that may be a useful resource for researchers studying the molecular mechanisms underlying prostate cancer development.

## Background

Gene-expression profiling has been extensively used to classify cancers by gene-expression signatures [1-3]. It has also been used for predicting response to treatment [4-6] and prognosis [7-9].

Ample gene-expression data are publicly available. Two major databases are Oncomine http://www.oncomine.org and the Gene Expression Omnibus (GEO; http://www.ncbi.nlm.nih.gov/geo/). Oncomine is a rapidly growing compendium of more than 20,000 cancer transcriptomes. Version 3.6, as accessed on January 5, 2010, comprised 35 datasets related to different aspects of prostate tumorigenesis. The GEO database is part of the open National Center for Biotechnology Information (NCBI) resources. GEO, the largest publicly available repository of gene-expression data [10], was established in 2000 to house and distribute those data to researchers [11]. This resource, however, is not generally used efficiently because it is not obvious how to combine data from different sources. Meta-analysis has the potential to reduce the biases of individual studies and may identify a reproducible set of genes [12-14].

A tumor's ability to invade distant sites may develop during relatively early stages of tumorigenesis and may be associated with the gene-expression signature in normal tissue, although the genes associated with metastatic progression would be expected to have their highest level of up-regulation or down-regulation during the later stages. Therefore, it should be beneficial to use data from different stages of prostate tumorigenesis to identify genes associated with prostate cancer development. We hypothesized that combining the gene-expression data from studies targeting different stages of prostate tumorigenesis can reliably identify those genes associated with prostate cancer development.

## Methods

Our approach is based on the ideas that tumorigenesis is a continuous process and that the gene-expression signature underlying the development of bone metastases starts to form in the early stages of prostate tumorigenesis and becomes well developed in the later stages. We have assumed that combining meta-analyses of different steps of prostate carcinogenesis and assigning greater weight to more-specific data might improve identification of genes associated with prostate tumorigenesis, especially those involved in the development of bone metastases; therefore, we have up-weighted the third level of meta-analysis.

### First level of the meta-analysis

The tendency of prostate tumor to metastasize to bone is a hallmark of prostate tumorigenesis. Breast and lung cancer also often metastasize to bone, although not as frequently as prostate cancer does. Bone-metastasizing cancers may share a gene-expression signature that predisposes them to form bone metastases. On the basis of this hypothesis, the first level of our meta-analysis focused on comparing bone-metastasizing and non-bone metastasizing cancers. Bone-metastasizing cancers included breast and lung cancers. Non-bone metastasizing cancers included colorectal and ovarian cancers, which very rarely metastasize to bone. The studies we used are listed in Additional file 1; all of them were aimed at identifying genes differently expressed between normal tissue and localized tumor tissues.

For the meta-analysis, we used Rosenthal's extension of Stouffer's method [15]. The choice of the method was dictated by the available data: for many studies, the individual gene-expression data points were not available, although the *t *test results and corresponding p values were readily accessible. In *Z *score-based meta-analysis, the individual p value *p*_*ij *_of gene *i *in study *j *will be converted to deviate: *z*_*ij *_= *F*^-1^(1-*p*_*ij*_), where *F *is the standard normal cumulative distribution function. *Z *scores can then be summed across studies: $Z_{i}=\frac{\sum_{j=1}^{N}z_{ij}}{\sqrt{N}}$, where *Z*_*i *_is the combined *Z *score for the *i*th gene and *N *is the number of studies (datasets). The *Z *score is positive when the gene is up-regulated in a more adverse phenotype and negative when it is down-regulated in a more adverse phenotype. We used the information on the direction of the changes in gene expression (up- or down-regulated from the original studies). If the gene was down-regulated, we directly converted the p value into a *Z *score to produce negative *Z *values. If the gene was up-regulated, we used 1 - p to obtain corresponding positive *Z *values.

The number of studies varied for the different cancers; e.g., 4 studies were used for breast and 9 for lung cancer. To correct for the differences, we adjusted individual *Z *scores to make the average absolute values the same across all types of cancer. For example, the average of the absolute values of the *Z *scores for breast cancer is 1.87, and that for lung cancer is 2.85. That difference is likely a result of the difference in sample size, i.e., 4 vs. 9 studies. To correct for the difference in sample size, we needed to adjust the *Z *scores for each probe so that the average absolute *Z*s will be the same and equal to (1.87 + 2.85)/2 = 2.36. Then the adjustment factor will be 2.36/1.87 = 1.26 for breast and 2.36/2.85 = 0.83 for lung cancer. The adjustment ensured equal contributions of individual cancer types to the global *Z *scores. This adjustment guarantees that differences between bone-metastasizing and non-bone metastasizing cancers are not driven by a single cancer type (e.g., lung cancer). Similar adjustments were made for the datasets used in the second and third levels of the meta-analysis. The sample size in individual studies is also an important factor that directly influences the p value: assuming the same effect size, the p value will be smaller for studies that have a larger sample size. Since smaller p values are transformed to larger *Z *scores, larger studies contribute more strongly to the global *Z *score than smaller studies do.

We subtracted the *Z *scores for the non-bone metastasizing cancers from those for the bone-metastasizing cancers. This subtraction takes away the genes that are common in the transition from normal tissue to localized tumor in bone-metastasizing and non-bone metastasizing cancer and simultaneously allows identification of genes that are up-regulated or down-regulated in the bone-metastasizing cancers.

### Second level of the meta-analysis

Table 1 lists the datasets we used for our second-level meta-analysis. For each gene, we computed the average *Z *score by using the *Z *scores from our first-level meta-analysis and those from the comparison of normal prostate vs. localized prostate cancer.

**Table 1:** Studies used for the second and third levels of our meta-analysis: genes expressed differently in the transition from normal prostate and localized prostate cancer to metastatic disease (Oncomine datasets)

Primary author's name	PMID	Tissue type (no. of samples) Class 1 Class 2	Total no. of genes	Level
Dhanasekaran	11518967	Normal prostate (22) Primary prostate cancer (59)	9,956	2

Dhanasekaran_2	15548588	Normal adjacent prostate (12) Prostate cancer (25)	19,650	2

Holzbeierlein	14695335	Normal prostate (4) Prostate cancer (23)	5,854	2

Lapointe	14711987	Normal prostate (41) Prostate carcinoma (62)	19,116	2

Luo	11406537	Benign hyperplasia (9) Prostate carcinoma (16)	6,500	2

Nanni	16513839	Normal prostate (3) Prostate carcinoma (22)	22,283	2

Tomlins	17173048	Benign prostate (22) Prostate carcinoma (30)	19,355	2

Vanaja	12873976	Normal prostate (8) Prostate adenocarcinoma (27)	44,928	2

Varambally	16286247	Benign prostate (6) Prostate carcinoma (7)	54,675	2

Welsh	11507037	Normal prostate (9) Prostate carcinoma (25)	11,138	2

Yu	15254046	Normal prostate (23) Prostate carcinoma (64)	12,625	2

Dhanasekaran	11518967	Primary prostate cancer (59) Metastatic prostate cancer (20)	9,935	3

Dhanasekaran_2	15548588	Prostate cancer (25) Metastatic prostate cancer (6)	18,502	3

Tomlins	17173048	Prostate carcinoma (30) Metastatic prostate cancer (19)	19,337	3

Yu	15254046	Prostate carcinoma (64) Metastatic prostate cancer (25)	12,625	3

Vanaja	12873976	Prostate adenocarcinoma (27) Metastatic prostate cancer (5)	44,928	3

Holzbeierlein	14695335	Prostate cancer (23) Metastatic prostate cancer (9)	6,475	3

LaTulippe	12154061	Prostate carcinoma (23) Metastatic prostate cancer (9)	12,600	3

### Third level of the meta-analysis

Table 1 also lists the studies we used for meta-analysis of genes expressed differently in localized and primary metastatic prostate cancers. All datasets for localized vs. metastatic prostate cancer comparisons are from the Oncomine database. We followed Oncomine's definition of analysis that compared localized vs. metastatic samples. Localized samples were from primary prostate tumor, and metastatic samples, from metastatic tumors (bone, lung, liver, and others). For clinical details, see the original publications indicated in Table 1. We computed average *Z *scores by using the scores from the second-level meta-analysis and those from meta-analysis of gene expression in localized vs. metastatic prostate cancer.

### Functional annotation

For functional annotation, we used the Database for Annotation, Visualization, and Integrated Discovery (DAVID; http://david.abcc.ncifcrf.gov/) [16]. DAVID tests the null hypothesis that genes are uniformly distributed across pathways and biologic functions. P values characterize the strength of statistical evidence for clustering: the lower the p value, the stronger the evidence that the genes are overrepresented in a specific pathway. Because our previous analyses [17,18] demonstrated that separate functional annotation of up-regulated and down-regulated genes tends to produce lower p values than joint analysis does, we separately annotated the 250 top down-regulated and the 250 top up-regulated genes.

### Proof of principle: *Z *scores of the genes with published evidence of an association with prostate cancer development

If our approach is methodologically sound, relative *Z *scores for the known prostate cancer-related genes are expected to rise from the first level to the third level. We used 2 sets of known prostate cancer-related genes--those identified by the KnowledgeNet approach [19] and those identified in the Gene Ontology (GO; http://www.geneontology.org/) database--for the functions and pathways that are known to be involved in prostate tumorigenesis: androgen receptor signaling, tumor growth factor beta (TGF-β) signaling, components of the extracellular matrix, cell adhesion, the Wnt signaling pathway, and bone development. The genes from those pathways are listed in Additional files 2 and 3.

### In silico validation: primary tumors vs. distant prostate metastases

The results of 2 studies comparing gene expression in primary tumors and distant metastases were recently published (GEO dataset GDS2547) [20,21]. We estimated the overlap between the genes expressed differently in primary tumors vs. distant metastases and the candidate genes our analysis identified.

### Experimental validation of the candidates: clinically advanced prostate cancer vs. benign prostatic hypertrophy

We used in-house gene-expression data to further validate the candidates identified by our meta-analysis. The tissue samples were frozen surgical specimens obtained using a protocol approved by the institutional review board of The University of Texas MD Anderson Cancer Center. The samples were from men undergoing cystoprostatectomies (n = 6) and pelvic exenterations (n = 3) for clinically advanced prostate cancer (CAPC). Seven of those 9 patients had had metastasis at the time of surgery; the other 2 patients had developed metastatic disease shortly after surgery. As controls, we used prostate tissues from 5 men with benign prostatic hypertrophy (BPH).

The tumor tissue samples were macrodissected from the frozen sections, and the RNA was extracted by using a mirVana miRNA isolation kit from Ambion, Inc. (Austin, TX). Microarray experiments were carried out using whole-human genome oligoarrays with 44,000 60-mer probes (with 500 ng of total RNA starting material) according to the manufacturer's protocol (Agilent Technologies, Inc., Santa Clara, CA). Hybridized arrays were scanned with Agilent's dual laser-based scanner, as described previously [22].

After the quality control procedure, we had 36,549 probes for analysis. The gene-expression data were normalized by using the limma package in R language http://www.bioconductor.org. The number of unique genes was 24,829. The mean expression values were calculated across all spots on the condition that they were mapped to the same gene. A standard *t *test with equal variance was used for calculating p values for the comparison of the tumor and normal samples. The raw data were normalized within and between arrays by using the composite method of Yang *et al*. [23]. The false-discovery rate was computed by using Significance Analysis of Microarrays software http://www-stat.stanford.edu/~tibs/SAM/.

## Results

### Gene-expression profile of bone-metastasizing cancers (breast plus lung) is similar to that of prostate cancer

*Z *scores characterize both the direction (a positive score means that the gene is up-regulated) and the strength of statistical evidence (the higher the absolute value of the *Z *score, the stronger the evidence). We found that the *Z *scores derived from the comparison of normal and localized tumor tissue of bone-metastasizing cancers (breast plus lung) were much more strongly correlated with those of the prostate cancers (normal prostate vs. localized prostate tumor) than they were with those of the non-bone metastasizing cancers (colon plus ovarian): Pearson's correlation coefficients for breast vs. prostate cancer were 0.29, n = 9,824; for lung vs. prostate cancer, 0.36, n = 10,824; for colon vs. prostate cancer, 0.21, n = 12,756; and for ovarian vs. prostate cancer, 0.22, n = 11,984. The difference in correlation coefficients between any bone-metastasizing and any non-bone metastasizing cancer was significant (minimal *Z *test = 8.1, p < 10^-6^). This supports the ideas that breast and lung cancers have a gene-expression signature in common with that of prostate cancer.

### Three-level meta-analysis

**Figure 1:**
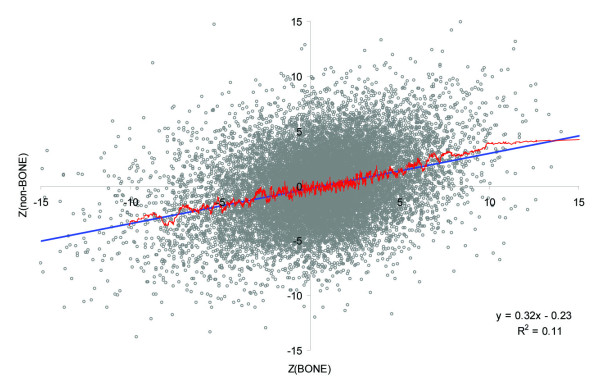
**Scatter plot of bone- and non-bone metastasizing cancers**. The blue line is a linear regression; the red line is a moving average computed for 100 adjacent genes.

The comparison of *Z *scores for bone-metastasizing and non-bone metastasizing cancers demonstrated a statistically significant positive Pearson's correlation between them: r = 0.33, n = 16,376, p < 10^-6^. Figure 1 is a scatter plot of the *Z *scores for bone-metastasizing and non-bone metastasizing cancers. It is evident from the plot that some genes are up-regulated in bone-metastasizing cancers and down-regulated in non-bone metastasizing cancers and vice versa. Those genes that are up-regulated in bone-metastasizing cancers might be specifically associated with the formation of bone metastases, which is a hallmark of prostate carcinogenesis. The genes identified in the first and second levels of our meta-analysis are listed in Additional files 4 and 5.

The genes and their corresponding *Z *scores from the third level of analysis are listed in Additional file 6. Functional annotation of down-regulated genes from the third level of the analysis identified cytoskeleton, cell adhesion, actin binding, and extracellular matrix as the top functional categories (Additional file 7). The associations between those genes and functions remained statistically significant after application of Bonferroni's adjustment for multiple testing. For the up-regulated genes, cell cycle and ATP binding were the most significant functional categories.

### *Z *score for known prostate cancer genes

The average absolute *Z *score for known prostate cancer genes was 2.06 ± 0.12, and the overall average score for the third level of the meta-analysis was 1.53 ± 0.01. The difference was statistically significant: *t *test = 6.03, df = 15,985, p < 10^-6^. Table 2 shows several pathways associated with prostate tumorigenesis. For all functional categories except cadherins and collagens, the average *Z *scores from the pathways were significantly higher than the overall average score. The most statistically significant differences were detected for the TGF-β signaling pathway, integrins, androgen receptor signaling pathway, extracellular matrix, and cell adhesion.

**Table 2:** Average absolute *Z *scores for genes from pathways and biologic functions associated with the development of bone metastases

**Function and gene category**^**a**^	**No. of genes**^**b**^	Absolute *Z *score (SE)	*t *test	Degrees of freedom	p value
TGF-β signaling	86	2.58 (0.34)	4.05	15,770	0.00005
Integrins	30	2.47 (0.35)	3.82	15,773	0.0001
AR signaling pathway	32	2.22 (0.34)	2.9	15,780	0.004
Components of EC matrix	64	2.11 (0.25)	3.41	15,812	0.0006
Cell adhesion	406	2.08 (0.09)	7.99	16,154	<10E-6
Wnt signaling pathway	29	2.06 (0.27)	2.13	15,777	0.03
Bone development	56	1.99 (0.24)	2.53	15,804	0.01
Collagens	40	1.76 (0.28)	1.07	15,759	0.28
Cadherins	23	1.35 (0.19)	0.61	15,767	0.54

Abbreviations: SE, standard error; TFG-β, transforming growth factor beta; AR, androgen receptor; EC, extracellular

^a^Functions were defined according to the Gene Ontology database.

^b^A list of genes for each pathway can be found in Additional file 2.

**Figure 2:**
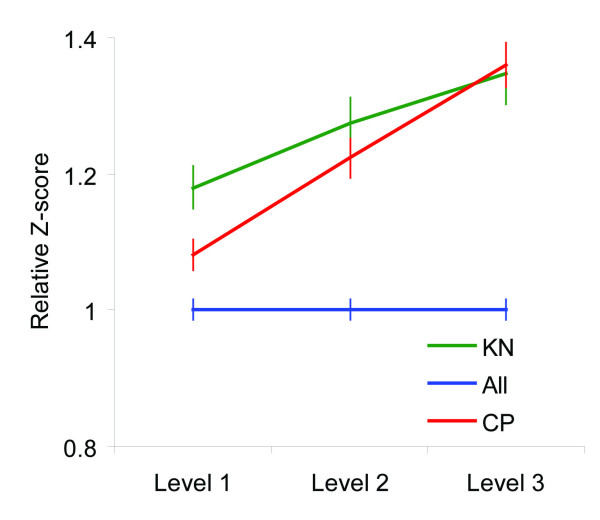
**Relative *Z *score for genes with published evidence of an association with the development of bone metastases based on the KnowledgeNet approach (KN) and for genes from candidate pathways (CP)**. Relative *Z *scores were computed as the ratio between the *Z *score for candidate genes and the overall average *Z *score. Vertical bars represent standard error (SE).

We separately computed average absolute *Z *scores for known prostate cancer genes in each of the 3 levels of meta-analysis. The genes identified through candidate pathways and by the KnowledgeNet approach were analyzed separately. For both groups, the relative *Z *scores increased significantly from the first to the third level of meta-analysis (Figure 2).

### Primary prostate tumor vs. distant metastasis

**Figure 3:**
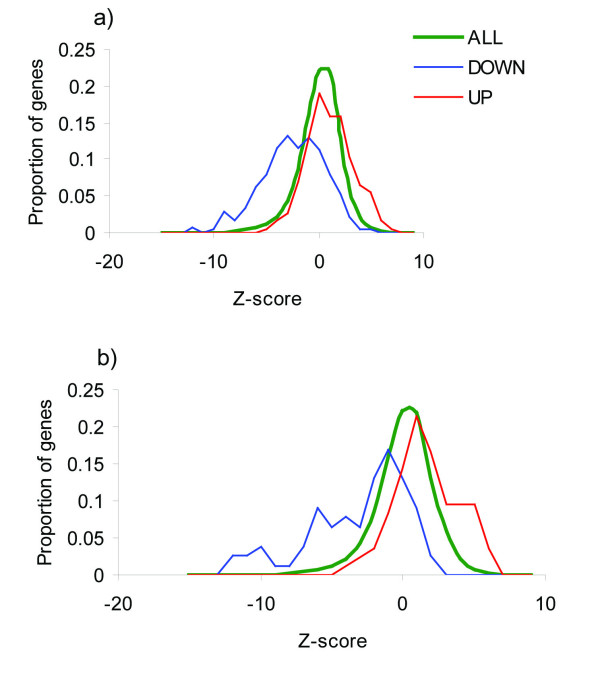
**Distributions of *Z *scores from the third-level analysis**. (*A*) The green line represents the distribution of all *Z *scores; the blue line, the distribution of *Z *scores for the genes found to be down-regulated in the study by Chandran *et al*. [20]; and the red line, the distribution of genes found to be up-regulated in Chandran's study. (*B*) Distribution of *Z *scores of genes found to be significantly up- and down-regulated between primary prostate tumor and bone metastases; study by Stanbrough *et al.*, 2006 [21].

The genes expressed differently between primary prostate tumor and distant metastases were derived from 2 recent studies [20,21]. Chandran *et al*. [20] compared gene expression in primary prostate tumor with that in distant non-bone metastases. Figure 3, *A*, shows the *Z*-score distribution of those genes. Most of the genes that were down-regulated in the distant metastases relative to the primary tumor also tended to be down-regulated in our analysis, and those up-regulated in distant metastases tended to be up-regulated in our analysis. The mean *Z *score for down-regulated genes was -2.49 ± 0.19, that for up-regulated genes was 1.02 ± 0.15, and the overall average was 0.08 ± 0.02. Similar results were obtained for the genes found to be differently expressed between primary tumors and bone metastases [21] (Figure 3B).

### Clinically advanced prostate cancer vs. benign prostatic hypertrophy

We used Spearman's correlation coefficient testing to assess for overall consistency between the results of the comparison of CAPC and BPH and the results of the third-level meta-analysis. The genes shared by the 2 studies were ranked on the basis of (a) *Z *scores from the third-level meta-analysis and (b) the log ratio of their expression values in the CAPC vs. BPH analysis. A positive significant correlation between the ranks was noted (r = 0.18, n = 15,503, p < 10^-6^).

## Discussion

Our meta-analysis integrated data from studies targeting a number of phenotypes related to prostate cancer development. The question is, does our meta-analysis perform better than analysis of a single phenotype, e.g., localized vs. metastatic primary tumors? It is impossible to answer this question directly because we do not have a "correct" list of genes associated with prostate cancer development. Indirect tests suggest that the 3-level meta-analysis does a better job of identifying candidate genes than the analysis of a single phenotype does. The top genes from our meta-analysis showed stronger functional clustering than did the top genes from individual meta-analyses. For example, the average -Log(p) value for the top 10 GO molecular functions identified by the 3-level meta-analysis was 4.1, whereas it was 1.9 in the comparison of localized and metastatic disease (the difference was statistically significant by the nonparametric sign test: *Z *= 2.9, p = 0.004). We also found that the relative *Z *score for the known prostate cancer genes increased from the first-level to the third-level meta-analysis. The top candidates from our meta-analysis strongly overlap with those genes that are (a) expressed differently in primary tumors and distant metastases, (b) involved in cancer development, and (c) expressed differently in CAPC and BPH. Taken together, these results suggest that our 3-level meta-analysis of the gene-expression data is an effective tool to identify the genes associated with prostate cancer development.

Recently Nakagawa *et al*. [24] published the results of a large retrospective case-control study of systemic progression of prostate cancer (GEO dataset ID GSE10645). Those authors identified 68 genes that are significantly up-regulated or down-regulated in patients with relapse defined by prostate-specific antigen concentration and systemic progression (detected on positive bone or computed-tomographic scanning) compared with those genes in patients with prostate-specific antigen concentration-defined relapse and no systemic progression. We found that our candidate genes overlapped with genes identified in Nakagawa's study: the correlation coefficient between the degree of change from Nakagawa's study and the *Z *scores from our analysis was 0.62, n = 58, p < 10^-6^. Genes that were significantly up-regulated after Bonferroni correction for multiple testing in both studies included *TOP2A, MKI67, CDC2, TPX2, SEC14L1, EIF2C2, THBS2, EZH2, CDKN3, BUB1, PGK1, CCNB1, HPRT1, MSR1, WDR67, CTHRC1, BIRC5, TAF2, YY1, RAD21, RAP2B, FAM49B, SQLE, F2R, CHRAC1, INHBA, SDHC*, and *NOX4*. Significantly down-regulated genes in both studies included *PAGE4*, *SRD5A2*, and *AZGP1*. We hypothesize that these 31 genes may be used to predict tumor progression after surgery.

One would expect that strong functional clustering of the top candidates might be a result of gene-gene interactions. To investigate this, we used Pathway Studio software [25] to construct the network of interactions of the top 200 genes. Only direct interactions--direct regulation of gene expression, protein-protein binding, or promoter binding--were used. We found that 96 genes formed the network of interactions. We then selected the genes that interacted with at least 5 other genes from the network and whose local connectivity was at least 2 times higher than the overall connectivity of the gene. This method identified 10 genes: *CDC2, CCNA2, IGF1, EGR1, SRF, CTGF, CCL2, CAV1, SMAD4*, and *AURKA*. Since those genes have a large number of downstream targets, they likely drive prostate cancer development and consequently are the best candidates for experimental verification.

A major limitation of this study is rooted in the available data and the fact that the genes assessed in the different studies only partially overlap. The studies we used were conducted for various purposes and, as a result, they are not absolutely homogeneous in terms of phenotype. This is especially relevant to the comparison of localized and metastatic cancer. Metastatic cancer included a variety of phenotypes possessing a gene-expression signature associated with the development of metastases. It is likely that the genes identified in our analysis do not completely reflect the diversity of genetic mechanisms underlying prostate cancer development but instead represent only the most common universal players in prostate tumorigenesis.

Another limitation is related to the fact that different studies use different platforms with the different sets of genes. Because of that, in our meta-analysis, different genes were assessed in a different number of the studies. One can expect a higher absolute *Z *score for a gene whose expression was assessed in a larger number of studies. In accord with this expectation, we found that the absolute *Z *scores positively correlated with the number of studies we used in the meta-analysis. However, our assessments demonstrate that this correlation is unlikely to explain more than 10% of the observed variation in *Z *scores (data not shown).

## Conclusions

In conclusion, our multilevel meta-analysis efficiently combined almost all publicly available gene-expression data on prostate cancer and allowed identification of candidate genes associated with prostate tumor development. The results of several *in silico *validation tests of the top candidate genes suggest that they are enriched by genes associated with prostate cancer development. The list of candidate genes we have generated may be a useful resource for researchers studying the molecular mechanisms underlying prostate cancer development.

## Abbreviations and acronyms

GEO: Gene Expression Omnibus database; GO: Gene Ontology database; TGF-β: transforming growth factor beta; CAPC: clinically advanced prostate cancer; BPH: benign prostatic hypertrophy;

## Competing interests

The authors declare that they have no competing interests.

## Authors' contributions

IPG, OYG, and CJL conceived the study; KS provided gene-expression data for validation; HZ and JYB performed the statistical analysis; and IPG wrote the manuscript with support from SNM, NMN, PT, and CAP. All authors read and approved the final manuscript.

## Pre-publication history

The pre-publication history for this paper can be accessed here:

http://www.biomedcentral.com/1471-2407/10/599/prepub

## Supplementary Material

Additional file 1 **Table S1**. Studies used in the first level of our meta-analysis: bone-metastasizing vs. non-bone metastasizing cancers (Oncomine datasets). Click here for file

Additional file 2 **Table S2**. List of the known candidate genes for bone metastasis identified by using the KnowledgeNet approach. Click here for file

Additional file 3 **Table S3**. Gene Ontology-defined genes from the pathways associated with development of bone metastasis. Click here for file

Additional file 4 **Table S4**. *Z *scores in the first level of the meta-analysis. Click here for file

Additional file 5 **Table S5**. *Z *scores in the second level of the meta-analysis. Click here for file

Additional file 6 **Table S6**. *Z *scores in the third level of the meta-analysis. Click here for file

Additional file 7 **Table S7**. Functional annotation of the top candidate genes from the level-3 meta-analysis. Click here for file
