# Supplementary material for: Prioritizing genes associated with prostate cancer development
Source: BMC Cancer. 2010 Nov 2;10:599. doi: 10.1186/1471-2407-10-599 (PMC2988752; doi:10.1186/1471-2407-10-599)
Supplement: Additional file 1 — Table S1. Studies used in the first level of our meta-analysis: bone-metastasizing vs. non-bone metastasizing cancers (Oncomine datasets). [file 1471-2407-10-599-S1.DOC]

**Table s1. Studies used in the first level of our meta-analysis: bone-metastasizing vs non–bone metastasizing cancers (Oncomine datasets)**

| Primary author’s name and cancer type | PMID | Tissue type (no. of samples) | No. of genes |
| --- | --- | --- | --- |
| Graudens_Colon | 16542501 | Normal colon (12) | 18,976 |
|  |  | Colorectal carcinoma (18) |  |
| Alon_Colon | 10359783 | Normal colon (22) | 1,988 |
|  |  | Colon adenocarcinoma (40) |  |
| Ki_Colon | 17640062 | Adjacent normal colon mucosa (28) | 15,783 |
|  |  | Primary colon carcinoma (53) |  |
| Zou_Colon | 12101425 | Normal colonic epithelium (8) | 7,486 |
|  |  | Invasive colon carcinoma (9) |  |
| Notterman_Colon | 11306497 | Normal colon (18) | 6,745 |
|  |  | Colon adenocarcinoma (18) |  |
| Hedrix_Ovarian | 16452189 | Normal ovary (4) | 22,283 |
|  |  | Ovarian serous adenocarcinoma (41) |  |
| Welsh_Ovarian | 11158614 | Normal ovary (4) | 4,995 |
|  |  | Ovarian adenocarcinoma (28) |  |
| Lancaster_Ovarian | 14706684 | Normal ovary (3) | 7,129 |
|  |  | Ovarian serous adenocarcinoma (31) |  |
| Adib_Ovarian | 14760385 | Normal ovary (4) | 12,625 |
|  |  | Ovarian adenocarcinoma(6) |  |
|  |  | Pancreatic adenocarcinima (12) |  |
| Finak_Breast | 18438415 | Normal breast (6) | 41,000 |
|  |  | Breast carcinoma (53) |  |
| Richardson_Breast_2 | 16473279 | Normal breast (7) | 54,613 |
|  |  | Breast carcinoma (40) |  |
| Sorlie_Breast | 11553815 | Benign breast (7) | 7,937 |
|  |  | Breast carcinoma (78) |  |
| Radvanyi_Breast | 16043716 | Normal breast (9) | 50,071 |
|  |  | Breast carcinoma (47) |  |
| Beer_Lung | 12118244 | Normal lung (10) | 6,856 |
|  |  | Lung adenocarcinoma (86) |  |
| Bhattacharjee_Lung | 11707567 | Normal lung (17) | 11,158 |
|  |  | Lung adenocarcinoma (139) |  |
| Bhattacharjee_Lung | 11707567 | Normal lung (17) | 10,881 |
|  |  | Small-cell lung cancer (6) |  |
| Stearman_Lung | 16314486 | Normal lung (19) | 12,625 |
|  |  | Lung adenocarcinoma (20) |  |
| Su_Lung | 17540040 | Normal lung (31) | 22,283 |
|  |  | Lung adenocarcinoma (31) |  |
| Garber_Lung | 11707590 | Normal lung (6) | 22,646 |
|  |  | Lung adenocarcinoma (40) |  |
| Wachi_Lung | 16188928 | Normal lung (5) | 22,215 |
|  |  | Squamous cell lung carcinoma (5) |  |
| Yamagata_Lung | 14581339 | Normal lung (3) | 3,709 |
|  |  | Squamous cell lung carcinoma (11) |  |
| Powell_Lung | 12600827 | Non-malignant lung (11) | 7,129 |
|  |  | Lung adenocarcinoma (11) |  |
