# Supplementary material for: Prioritizing genes associated with prostate cancer development
Source: BMC Cancer. 2010 Nov 2;10:599. doi: 10.1186/1471-2407-10-599 (PMC2988752; doi:10.1186/1471-2407-10-599)
Supplement: Additional file 3 — Table S3. Gene Ontology-defined genes from the pathways associated with development of bone metastasis. [file 1471-2407-10-599-S3.DOC]

**Table S3.**

**GO-defined genes from the pathways associated with development of bone metastasis.**

Androgen Signaling:

AR, BRCA1, MED17, PIAS2, DAXX, MED1, MED24, CCNE1, GRIP1, ARID1A, PPARGC1A, NCOA3, MED13, MED30, NRIP1, RB1, RNF4, RAN, NCOA1, PPAP2A, THRAP3, MED12, MED14, RNF14, CDK7, MED16, PIAS1, NCOA4, CTNNB1, MED4, FHL2, TGFB1I1,

Bone Development

BMP6, ENPP1, IL6R, CASR, ENAM, MEN1, BMP2, OSTF1, SOST, TUFT1, BMPR1B, P2RX7, ACHE, EIF2AK3, CDK6, BGLAP, ATP6V1B1, ATP6V0A4, ACVR2B, AMBN, ZNF675, EXT1, DMP1, MINPP1, DSPP, AMELX, RUNX2, GLI1, CYP24A1, CDH11, AHSG, NF1, CYP27B1, KL, BMP7, BMPR2, GLI2, SPARC, STATH, COL13A1, EGFR, TWIST2, IL6, TGFB1, TGFB3, ACVR2A, BMP4, SBDS, SRGN, EXT2, TGFB2, IL6ST, ACVR1, ANKH, MGP, BMPR1A

Wnt Signaling

NLK, CD24, PPM1A, AXIN1, GSK3B, CXXC4, TLE1, HHEX, DVL1, LRP6, MDFI, SENP2, CHD8, TCF7L1, RARG, UBE2B, FRZB, APC, DKK4, CELSR2, PPP2CA, MDFIC, ZRANB1, PPP2R1A, CITED1, PTPRU, TCF7L2, CTNNB1, TAX1BP3

Components of Extracellular Network

ACHE, ADAMTS13, ADAMTS3, ADAMTS5, ADAMTS9, AMBN, AMELY, APLP1, CD248, CHI3L1, COL13A1, COL14A1, COL16A1, COL19A1, COL4A4, COL4A6, COL6A3, COL7A1, COL8A1, COL8A2, COL9A1, COL9A2, COL9A3, CRISP3, DGCR6, DST, ECM1, ECM2, EFEMP2, ENAM, EPB41L2, ERBB2IP, FBLN2, FBLN5, FBN2, FLRT1, FLRT2, FLRT3, FMOD, IMPG1, K222, KAL1, KERA, LTBP2, MEPE, MFAP1, MFAP4, MMP10, MMP11, MMP19, MMP23A, MMP25, MMP26, MMP28, MTM1, MUC4, MUC5AC, mucin, O43266, OPTC, PI3, PRSS2, Q9H3W0, SCUBE3, SNCA, SPOCK2, TFPI2, THBS1, TNXB

Cell Adhesion

ACHE, ACTN1, ACTN2, ACTN3, ACVRL1, ADA, ADAM10, ADAM15, ADAM17, ADAM22, ADAM9, ADAMDEC1, ADAMTS13, ADIPOQ, AGGF1, ALCAM, ALOX12, ALX1, AMBN, AMBP, AMELX, AMIGO1, AMIGO2, AMIGO3, ANGPTL3, ANXA9, AOC3, APBA1, APC, APOA4, ARF6, ARHGAP5, ARHGAP6, ARHGDIA, ARHGDIB, ARHGDIG, ARVCF, ASTN1, ATP2A2, ATP2C1, AZGP1, AZU1, B4GALNT2, BAI1, BCAM, BCL10, BGLAP, BMP1, BMPR1B, BYSL, C9orf127, CADM1, CADM3, CALCA, CASK, CCL11, CCL2, CCL4, CCL5, CCR1, CCR3, CCR8, CD151, CD164, CD2, CD209, CD22, CD226, CD33, CD34, CD36, CD40LG, CD44, CD47, CD58, CD72, CD84, CD9, CD93, CD96, CD97, CDH1, CDH10, CDH11, CDH12, CDH13, CDH15, CDH16, CDH17, CDH19, CDH2, CDH20, CDH23, CDH24, CDH4, CDH5, CDH6, CDH7, CDH8, CDH9, CDK5R1, CDK6, CDKN2A, CDON, CDSN, CEACAM1, CELSR1, CELSR2, CERCAM, CHL1, CHRD, CHST10, CHST4, CIB1, CITED2, CLDN1, CLDN10, CLDN11, CLDN12, CLDN14, CLDN15, CLDN16, CLDN17, CLDN18, CLDN19, CLDN2, CLDN20, CLDN22, CLDN23, CLDN3, CLDN4, CLDN5, CLDN6, CLDN7, CLDN8, CLDN9, CLEC4A, CLEC4M, CNTN1, CNTN2, CNTN4, CNTN6, COL13A1, COL14A1, COL15A1, COL16A1, COL17A1, COL19A1, COL3A1, COL5A1, COL6A1, COL6A2, COL8A2, CORO1A, CRNN, CSF1, CTNNA1, CTNNA2, CTNNA3, CTNND1, CTNND2, CUZD1, CX3CL1, CX3CR1, CXCL12, CXCR3, CYFIP2, CYTH1, CYTIP, DCHS1, DDR1, DDR2, DEFB118, DGCR6, DLC1, DLG1, DLG5, DLL1, DPP4, DSC2, DSC3, DSCAM, DSG1, ECM2, EFNB1, EGFR, EMCN, EMILIN1, EMR1, ENG, ENTPD1, ERBB2, ERBB2IP, ERBB3, EZR, FBLN5, FERMT2, FEZ1, FLOT2, FN1, FPR2, FXC1, GMDS, GNE, GP1BA, GP5, GP9, GPR56, GPR98, GTPBP4, HABP2, HAS1, ICAM1, IGFBP7, IL12A, IL12B, IL18, IL32, IL8, ILK, ISLR, ITGA1, ITGA10, ITGA11, ITGA2, ITGA2B, ITGA3, ITGA4, ITGA5, ITGA7, ITGA8, ITGA9, ITGAD, ITGAL, ITGAM, ITGAV, ITGAX, ITGB1, ITGB1BP1, ITGB2, ITGB3, ITGB3BP, ITGB4, ITGB6, ITGB7, ITGB8, ITGBL1, IZUMO1, JAM2, KAL1, KIRREL2, KNG1, L1CAM, LAMA3, LAMB1, LAMC1, LGALS4, LGALS7, LMO4, LOXL2, LPXN, LRRN2, LY6D, LYVE1, MADCAM1, MAEA, MAGI1, MCAM, ME2, MFAP4, MFGE8, MGP, MIA3, MLLT4, MMRN1, MPZL2, MSLN, MSN, MTSS1, MUC16, MUC5AC, MUC5B, MUPCDH, NCAM1, NCAM2, NEDD9, NELL2, NEO1, NF1, NF2, NID2, NINJ1, NINJ2, NLGN1, NLGN2, NME2, NPHP1, NPHP4, NPHS1, NPTN, NRCAM, NRG1, NRP2, OPCML, PARVG, PCDH24, PCDHA1, PCDHA10, PCDHA11, PCDHA2, PCDHA3, PCDHA4, PCDHA5, PCDHA6, PCDHA7, PCDHA8, PCDHAC1, PCDHAC2, PCDHB10, PCDHB11, PCDHB12, PCDHB13, PCDHB14, PCDHB15, PCDHB16, PCDHB2, PCDHB3, PCDHB4, PCDHB5, PCDHB6, PCDHB7, PCDHB9, PCDHGB4, PCDHGC3, PKD1, PKD1L1, PKD2, PKHD1, PKP1, PKP2, PKP4, PLXNC1, PNN, POSTN, PPFIA1, PPFIA2, PPFIBP1, PPP2CA, PPP2R1A, PRR3, PRSS2, PSEN1, PSTPIP1, PTEN, PTPRF, PTPRK, PTPRM, PTPRT, PTPRU, PVRL1, PVRL2, PXN, RAB13, RAC1, RASA1, REG3A, RGMB, RHOB, RND1, RND3, ROBO1, ROBO2, ROCK1, ROPN1B, RPSA, RS1, SAA1, SELL, SELP, SELPLG, SEMA4D, SEMA5A, SGCE, SIGLEC1, SIPA1, SIRPA, SIRPG, SLAMF7, SLURP1, SORBS1, SPN, SPOCK1, SSPN, STAB1, STAB2, SYK, TAOK2, TESK2, TGFB1I1, TGFB2, TGFBI, TGM2, THBS1, THBS3, THY1, TINAG, TLN2, TNC, TNF, TNR, TNXB, TPBG, TPM1, TRIP6, TRO, TROAP, TSC1, TSC2, TSTA3, TYRO3, VANGL2, VCAM1, VCAN, VCL, VTN, VWF, ZAN, ZYX

TGFB Signaling

CHRD, NOG, COMP, THBS1, THBS2, THBS3, THBS4, DCN, FST, BMP2, BMP4, BMP8A, BMP5, BMP6, BMP7, BMP8B, GDF7, GDF6, GDF5, AMH, TGFB1, TGFB2, TGFB3, NODAL, INHBA, INHBB, INHBC, INHBE, LEFTY1, LEFTY2, ACVR2A, ACVR2B, BMPR2, TGFBR2, AMHR2, BMPR1A, BMPR1B, ACVR1C, TGFBR1, ACVR1B, ACVR1, ACVRL1, SMAD1, SMAD5, SMAD9, SMAD2, SMAD3, SMAD4, SMAD6, SMAD7, SMURF1, SMURF2, ZFYVE9, ZFYVE16, ID1, ID2, ID3, ID4, RBL1, RBL2, E2F4, E2F5, TFDP1, CREBBP, EP300, SP1, MYC, CDKN2B, PITX2, RBX1, CUL1, SKP1, [KO:K03094], MAPK1, MAPK3, IFNG, TNF, RHOA, ROCK1, ROCK2, RPS6KB1, RPS6KB2, PPP2R1A, PPP2R1B, PPP2CA, PPP2CB, LTBP1

Integrins

ITFG2, ITGB1BP1, ITGB3, ITGB2, ITGB1, ITGAV, ITGA5, ITGA3, ITGA2, ITGA6, ITGB5, ITGA4, ITGA2B, ITGB4, ITGAL, ITGA11, ITGAM, ITGA1, ITGA10, ITGAE, ITGAX, ITGA7, ITGB7, ITGB6, ITGA9, ITGA8, ITGAD

Cadherins

CDH1, CDH10, CDH11, CDH12, CDH13, CDH15, CDH16, CDH17, CDH18, CDH19, CDH2, CDH20, CDH22, CDH23, CDH24, CDH26, CDH3, CDH4, CDH5, CDH6, CDH7, CDH8, CDH9

Collagens

COL10A1, COL11A1, COL11A2, COL11A2P, COL12A1, COL13A1, COL14A1, COL15A1, COL16A1, COL17A1, COL18A1, COL19A1, COL1A1, COL1A2, COL20A1, COL21A1, COL22A1, COL23A1, COL24A1, COL25A1, COL27A1, COL28A1, COL29A1, COL2A1, COL3A1, COL4A1, COL4A2, COL4A3, COL4A3BP, COL4A4, COL4A5, COL4A6, COL5A1, COL5A2, COL5A3, COL6A1, COL6A2, COL6A3, COL6A6, COL7A1, COL8A1, COL8A2, COL9A1, COL9A2, COL9A3, COLQ,
